# The Siamese Teeth

**Published:** 1879-08

**Authors:** 


### Editorial.

### THE SIAMESE TEETH.

A correspondent in Siam writes as follows of the teeth of the nations of that country:

"I have had a good deal to do with the teeth of the Siamese princes and noblemen. Several of the king's brothers have been under my hands. They all have black teeth from the use of the betel root, which they place in a leaf of the tree, mixed with lime. The effect is exhilerating almost to intoxication, especially to those not accustomed to its use. It leaves a deposit on the teeth which prevents decay, but it destroys the parts about the teeth, so that seldom will a person be found over forty years of age who has teeth firm in the sockets, and a great many have lost every tooth in the mouth from this cause solely."

Well, this is keeping pretty well abreast with civilized and cultered Americans, many of whom, by the use of tobacco and various kinds of filth, render the teeth more unsightly and disgusting than if they were ebony black. And as to the loss of teeth, even to the entire dentures, no heathen nation on earth can equal the goodly people of the United States.

Apologetically, however, it may be said that the latter have been subjected to a system of vandalism by charlatans that no savage nation ever experienced or would have permitted. But a better day is dawning.

